# Functional investigation of bone implant viability using radiotracers in a new model of osteonecrosis

**DOI:** 10.6061/clinics/2016(10)11

**Published:** 2016-10

**Authors:** Luis Schiper, Bluma Linkowski Faintuch, Roberto José da Silva Badaró, Erica Aparecida de Oliveira, Victor E. Arana Chavez, Elisangela Chinen, Joel Faintuch

**Affiliations:** I Universidade Federal da Bahia, Faculdade de Medicina, Departamento de Ortopedia, Bahia/BA, Brazil; II Instituto de Energia e Pesquisa Nuclear, Centro de Radiofarmácia, São Paulo/SP, Brazil; III Universidade de São Paulo, Faculdade de Odontologia, Departamento de Biomateriais e Biologia Oral, São Paulo/SP, Brazil; IV Hospital das Clínicas da Faculdade de Medicina da Universidade de São Paulo, Divisão de Cirurgia Gastrointestinal, São Paulo/SP, Brazil

**Keywords:** Osteonecrosis, Angiogenesis, Femur, Abdominal Pocket, Radiotracers, Technetium-99m, Experimental Model

## Abstract

**OBJECTIVES:**

Conventional imaging methods are excellent for the morphological characterization of the consequences of osteonecrosis; however, only specialized techniques have been considered useful for obtaining functional information. To explore the affinity of radiotracers for severely devascularized bone, a new mouse model of isolated femur implanted in a subcutaneous abdominal pocket was devised. To maintain animal mobility and longevity, the femur was harvested from syngeneic donors. Two technetium-99m-labeled tracers targeting angiogenesis and bone matrix were selected.

**METHODS:**

Medronic acid and a homodimer peptide conjugated with RGDfK were radiolabeled with technetium-99m, and biodistribution was evaluated in *Swiss* mice. The grafted and control femurs were evaluated after 15, 30 and 60 days, including computed tomography (CT) and histological analysis**.**

**RESULTS:**

Radiolabeling achieved high (>95%) radiochemical purity. The biodistribution confirmed good blood clearance 1 hour after administration. For ^99m^Tc-hydrazinonicotinic acid (HYNIC)-E-[c(RGDfK)_2_, remarkable renal excretion was observed compared to ^99m^Tc-methylene diphosphonate (MDP), but the latter, as expected, revealed higher bone uptake. The results obtained in the control femur were equal at all time points. In the implanted femur, ^99m^Tc-HYNIC-E-[c(RGDfK)_2_ uptake was highest after 15 days, consistent with early angiogenesis. Regarding ^99m^Tc-MDP in the implant, similar uptake was documented at all time points, consistent with sustained bone viability; however, the uptake was lower than that detected in the control femur, as confirmed by histology.

**CONCLUSIONS:**

1) Graft viability was successfully diagnosed using radiotracers in severely ischemic bone at all time points. 2) Analogously, indirect information about angiogenesis could be gathered using ^999m^Tc-HYNIC-E-[c(RGDfK)_2_. 3) These techniques appear promising and warrant further studies to determine their potential clinical applications.

## INTRODUCTION

Avascular bone ischemia and necrosis encompass a cluster of orthopedic phenomena, which can affect multiple bones in different settings. From unrecognized microfractures and necrotic foci to total bone collapse and loss of function, these conditions can lead to a variety of clinical courses. The idiopathic modality is the most studied, especially in subarticular (subchondral) regions of the long bones, such as classic Calve-Legg-Perthes disease of the head of the femur. Bone infarcts can also occur secondary to embolisms of defective red blood cell agglomerates, typical of sickle cell conditions, and those associated with blood clots, fats and gas embolisms 1,2.

Virtually any type of bone can be affected by such precipitants, which also include trauma (fractures and dislocations), infections, alcoholism, rheumatologic disorders (e.g., lupus), radiotherapy (osteoradionecrosis), and the use of certain drugs, particularly corticosteroids 2,2.

Moreover, these conditions are concerning when bone grafts are therapeutically used to resolve clinical situations with loss of bone substance. The viability of the transferred graft plays a critical role in the mechanical and physiological properties of the bone fragment.

For example, temporary ischemia of large bone segments with subsequent reimplantation can occur after decompressive craniectomy to treat brain swelling. Depending on the severity of the intracranial hypertension, primary closure of the skull might become impossible, in which case, the flaps must be stored until cranioplasty becomes feasible. Placement in a subcutaneous pocket of the lower abdominal wall for weeks or up to a month or more represents an alternative method of preserving the bone flaps 3.

Large, unresectable chondrosarcomas and other tumors of the calvaria, mandible and femur can rarely be extracorporeally treated by autoclavation or other cancer sterilization procedures, followed by reimplantation. Although the removed bone does not require prolonged storage and can be orthotopically replaced fairly promptly, the graft becomes totally devitalized, and subsequent histological reactions can occur 4.

Experimental animal models of osteonecrosis are not lacking, and an array of ischemic, traumatic and pharmacologic induction options have become available 5,6,7. Most have been aimed at Calve Legg Perthes disease and, thus, require very detailed and cumbersome techniques 8. Disability is intrinsic to such models, as seen in the human context; however, these approaches can shorten survival and hamper the long-term observation of the animals.

In the present protocol, a reliable, inexpensive, and easily reproducible option was sought. Such a model should reflect the general bone shifts that occur over the course of prolonged total ischemia, not only those germane to aseptic necrosis of the head of the femur. Freely moving animals should also be included in the design to minimize the long-term mortality triggered by partial or total immobilization.

Ideally, the model should provide a stable substrate for the functional assessment of prolonged major bone ischemia. Standard imaging methods, including computed tomography (CT) and magnetic resonance imaging (MRI), are excellent for diagnosing the established clinical consequences of bone damage. However, their roles in functional follow-up—namely, to determine the progression or regression of bone viability—have not been demonstrated 1,7,8.

Biphosphonates radiolabeled with the radioisotope technetium-99m (^99m^Tc) 9,10 have been used as general bone diagnostic imaging agents for decades. Therefore, they were a logical choice for the assessment of bone function within the context of this ischemia model. Angiogenesis, a functional process involved in bone regeneration, includes many mediators, such as integrins, and the tripeptide RGD sequence is a target amino acid recognition sequence for many of these mediators 11.

Evidence of angiogenesis was also within the scope of this investigation because this phenomenon is directly pertinent to bone viability, although molecular confirmation of the involved radionuclide mediators and receptors was outside the scope of this protocol.

Under these circumstances, a new model employing whole femur grafts syngeneically implanted in subcutaneous pouches in mice was devised and monitored for 60 days by two different radiotracers: ^99m^Tc-MDP and ^99m^Tc-HYNIC-E-[c(RGDfK)_2_.

The objective of this study was the longitudinal analysis of large bone avascular necrosis in mobile animals. It was hypothesized that a stable, reproducible and convenient experiment would be achieved and that bone viability would be successfully detected by systemically injected isotopes, despite the constraint imposed by a lack of vascularization. To the best of our knowledge, this model of osteonecrosis has not been reported before, and this study represents the first assessment of the radiotracer imaging technique 12.

## MATERIALS AND METHODS

### Compliance with ethical standards

The study was approved by the Institutional Animal Welfare Committee of the Instituto de Pesquisas Energéticas e Nucleares (IPEN)/Comissão Nacional de Energia Nuclear (CNEN), São Paulo, SP, Brazil, and both the “Principles of laboratory animal care" (NIH publication 8023, revised 1978 and 86-23, revised 1985) and the national guidelines for animal experimentation, as established by the Scientific Ethics Committee, Institute of Energy and Nuclear Research, National Nuclear Energy Committee, Brazil, and the Brazilian College of Animal Investigation, were followed.

### Materials

**Figure 1:**
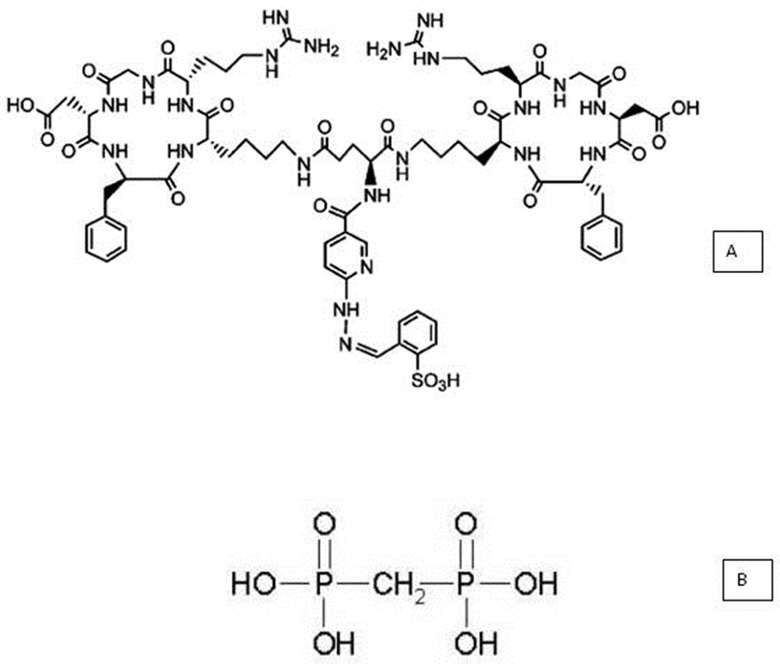
Structure of conjugated HYNIC-E[c(RGDfK)]_2_ (A) and methylene diphosphonic acid (MDP) (B).

- The cyclic homodimer peptide-conjugated arginine-glycine-aspartic acid and D-phenylalanine-lysine analog, with glutamic acid connecting the dimers [abbreviated (HYNIC-E-[c(RGDfK)_2_)] (MW 1453.6, peptide purity: 95%), was purchased from CPC Scientific Inc. (Sunnyvale, CA, USA) as lyophilized powder (Figure 1A). The conjugate was dissolved in sterile water at a concentration of 687.94 µM and was stored at -20°C.
- A lyophilized medronic acid kit consisting of 10 mg of medronic acid (Figure 1B), 1.2 mg of dehydrated stannous chloride, and 2 mg of paraminobenzoic acid (produced in house) was used (Radiopharmacy Center, Institute of Energy and Nuclear Research/National Commission of Nuclear Energy-IPEN/CNEN, São Paulo, SP, Brazil).
- Technetium-99m (^99m^Tc), in the form of Na^99m^TcO4, was eluted in saline from an alumina-based ^99^Mo/^99m^Tc generator, which was supplied by the same radiopharmacy center (IPEN/CNEN, São Paulo, SP, Brazil).
- Silica gel strips (SGs) for instant thin-layer chromatography (ITLC) (5 × 20 cm; Pall Corporation, NY, USA) and chromatography paper (Whatman 3 MM; Fisher Scientific, Loughborough, UK) were also used.
- Reversed-phase high-performance liquid chromatography (RP-HPLC, Shimadzu, Kyoto, Japan) was performed using a Symmetry C-18 column (5.0 mm, 100 A°, 4.66250 mm, Waters, Milford, MA, USA). Radioactivity measurements were collected using an automated, well-type c-counter NaI(Tl) crystal (Canberra, Meriden, CT, USA).

### Radiotracer preparation

#### Radiolabeling of MDP with technetium-99m

The freeze-dried kit was reconstituted using 3 mL of freshly eluted Na^99m^TcO4 in a sterile physiological saline solution with an activity of 30 mCi (1,110 MBq). The vial was stirred and maintained at room temperature for 15 min to allow the reaction to proceed.

### HYNIC-E-c(RGD)_2_ labeling with ^99m^Tc using ethylenediamine-N,Nʹ-diacetic acid (EDDA)/tricine as exchange products

The labeling procedure was performed as previously described by our group 13.To a sealed reaction vial containing 20 mg of tricine and 5 mg of EDDA, 0.5 mL of previously nitrogenated 0.1 M phosphate-buffered saline was added to dissolve the salts. Then, 10 μl of 687.94 µM HYNIC-E-c(RGDfK)_2_ plus stannous chloride (SnCl_2_^.^H_2_O) in 0.1 N HCl (nitrogen-purged) and 500 μL of Na^99m^TcO_4_^-^ (1850 MBq) were added. The vial was heated for 20 minutes at 100°C and then cooled to room temperature. The pH of the reaction was 7.

### Radiochemical control

The radiochemical evaluation of ^99m^Tc-MDP was performed by ascending paper chromatography using Whatman 3 MM chromatography paper as the support; the two solvent systems consisted of acetone and 0.9% sodium chloride solution.

In contrast, ITLC-SG was used for the radiochemical evaluation of ^99m^Tc-HYNIC-E-[c(RGDfK)_2_ with a two solvent system consisting of methylethylketone and 50% acetonitrile. The ITLC results were confirmed by RP-HPLC using a C18 column (5.0 mm, 100A°, 4.6 x 250 mm) and a flow rate of 1.0 mL/min. The solvent system consisted of H_2_O containing 0.1% trifluoroacetic acid (solvent A) and acetonitrile containing 0.1% trifluoroacetic acid (solvent B). The HPLC gradient system began with a solvent composition of 95% A and 5% B, followed by a linear gradient to 30% A:70% B from 0 to 25 min and, then, 5% A:95% B from 25 to 30 min.

### Study end points

The primary end point was the successful uptake of the two radiomarkers by totally devascularized bone grafts, compatible with the vitality of the subcutaneously housed ischemic bone. The secondary end point was the analysis of the technical difficulties of the model and the radionuclide procedure to gain insight into their future utility.

### Biological studies and study samples

All *in vivo* studies were performed in young female inbred Swiss mice (8 weeks old) with weights of approximately 20 g. Females were selected because they were more easily available. Age and weight were defined for ease of handling while excluding any age-related osteoporosis. Because this study was the first of its kind and no previous reports are available in the literature, formal sample size estimation could not be performed. However, given the syngeneic features and the homogeneous weight, age and sex of the model, 6 mice for each isotope and observation time were considered adequate to obtain statistically relevant findings. The studied population was 54 mice divided into three groups (one for each isotope and one for general purposes, such as tomographic imaging, macroscopic evaluation, and histological control), with 6 animals analyzed at each of the three time points. Another 27 animals were used as femur donors (two femurs from each donor), for a total of 81 mice. All animals were sacrificed at the end of the corresponding observation period and analyzed according to the protocol. The exceptions were, naturally, the donors, which were all sacrificed at the beginning of the investigation**.** The animals were provided by the Animal Facility of IPEN/CNEN, São Paulo, SP, Brazil.

### Housing and handling of the animals

The mice were maintained in individual cages under a 12-h/12-h light/dark cycle and pathogen-controlled conditions. They had *ad libitum* access to water and standard rodent chow. All of the procedures were performed in the morning in the technical laboratory. Radioactive materials, including the sacrificed animals, were quarantined until isotope decay and then discarded in accordance with international directives (International Atomic Energy Agency).

### Biodistribution evaluation

The animals were sacrificed by anesthetic drug overdose 1 h after the administration of the radiotracer. Tissues and organs were excised and weighed, and the radioactivity was measured with a gamma counter (Cobra 5002, Packard, USA), using the injected dose as a standard for calculation. The results were expressed as the percentage of the injected dose per gram (%ID/g).

### Subcutaneous femur implantation

Donor animals were sacrificed by anesthetic overdose, and their femurs were surgically excised. The homolateral femurs were subcutaneously grafted into a surgically created abdominal pouch. The animals were anesthetized (1 mL/kg IP ketamine and 0.5 ml/kg IP xylazine) and shaved for the surgical intervention.

A transverse skin incision was made on the right side of the abdomen between the last rib and the hip. A pocket was created for the implantation of the femoral graft. After the femur was successfully placed, the incision was sutured. No skin mark was necessary because the bone was easily palpable.

The mice were allowed to recover with food and water. The water supplied during the first 5 days contained an antibiotic (oxacillin) and an analgesic (acetaminophen).

### Tomographic imaging

Whole-body tomographic images were acquired in a Preclinical System PET/SPECT/CT (Bruker, Billerica, MA, USA). For technical reasons, the follow-up times were reduced to only two: baseline (bone implantation) and the end of the experiment (60 days).

### Radiotracer uptake in grafted vs. control femurs

After 15, 30 and 60 days, radiotracers were injected. For the acquisition of images, the mice were anesthetized and horizontally placed under the low-energy, high-resolution (LEHR) collimator of a Mediso Imaging System (Budapest, Hungary). Images were acquired 3 min before sacrifice using a 256 x 256 x 16 matrix size with a 20% energy window set at 140 keV for a period of 180 seconds.

The animals were then sacrificed. Both the grafted and orthotopic homolateral control femurs were excised and weighed, and the radioactivity was measured using a gamma counter as described in item 4.

Grafted and control femurs were also created and harvested without the administration of radiotracers during the same periods for histological analysis.

### Macroscopic images of the specimens

Representative bone grafts were photographed at different times for illustrative purposes. Because a hand-held apparatus and not photometric equipment or software was used, minor imaging distortions may have occurred.

### Histological evaluation

The excised femurs were fixed in buffered formalin solution. Following decalcification for 30 days, they were embedded in paraffin blocks. Representative slides from mid-diaphysis and the subchondral areas (for comparison) were then processed for routine hematoxylin and eosin staining.

### Statistical analysis

The radiolabeling results, tomographic images and histological sections were interpreted by professionals blinded to the group and the timing of the experiments.

The results are presented as percentages or the means ± standard deviations. Numerical findings were compared using Student’s “t” test or analysis of variance using the Statistical Package SPSS 17.0 (2012). Differences were considered significant when *p*<0.05.

## RESULTS

The radiolabeling of both molecules with ^99m^Tc resulted in high radiochemical purity. The labeling efficiency for ^99m^Tc-MDP was assessed by paper chromatography using a solvent system and was found to be 97.42±0.89%. In both solvents (acetone and 0.9% sodium chloride solution), the impurity pertechnetate (^99m^TcO_4_) had a retention factor (Rf) of 1, whereas the colloid ^99m^TcO_2_ impurity was quantified as Rf=0. The radiolabeled ^99m^Tc-MDP in the former solvent remained with the colloid. In the latter solvent, the Rf was the same as that for pertechnetate.

The radiochromatogram acquired via HPLC for ^99m^Tc-HYNIC-E-c(RGDfK)_2_ demonstrated a single peak at 14.83 min, with a radiochemical purity of 98.85±0.23%.

The amount of the colloid (^99m^TcO_2_) was only detectable by ITLC-SG and was found to be approximately 0.13%.

The biodistribution of radiotracers revealed good blood clearance. Because the ^99m^Tc-HYNIC-E-[c(RGDfK)_2_ conjugate was relatively hydrophilic, remarkable renal excretion was observed compared to ^99m^Tc-MDP.

Hepato-intestinal excretion also occurred, whereas with ^99m^Tc-MDP, only hepatic excretion was noted, and the enteric concentrations were very low. The uptake by the spleen was similar. ^99m^Tc-MDP exhibited much higher affinity for bone than ^99m^Tc-HYNIC-E-c(RGDfK)_2_, as expected.

**Figure 2:**
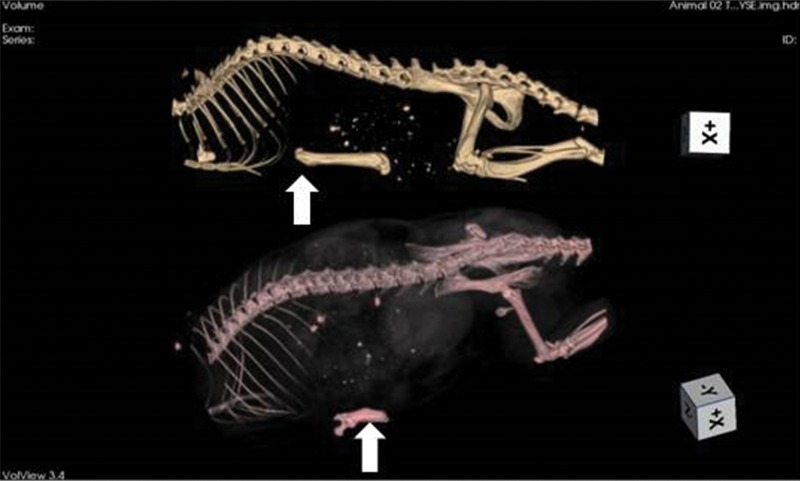
Tomographic imaging of an animal immediately after syngeneic femur implant (upper image) and at 60 days of storage (lower image). The grafts (arrows) can be identified below the rib cage. On the 60-day image, the bone resorption is highly advanced (nearly 50% of the mass). This phenomenon did not always occur, with other animals exhibiting far less bone loss.

Shortly after femur grafting (baseline), tomographic imaging was performed (Figure 2).

After each scheduled follow-up period (15, 30 and 60 days), radiotracers were injected for bone assessment. *In vivo* images were acquired, and subsequently, the removed bone was examined for tracer uptake and compared to the control femur. Nonradioactive grafts and orthotopic homolateral controls were surgically excised simultaneously for histological analysis. After 60 days, as mentioned above, CT imaging was also performed for comparison with baseline findings.

Surgical bone removal revealed major changes in the size, texture, bone integrity, and surrounding tissues, depending on the timing of the procedure.

**Figure 3:**
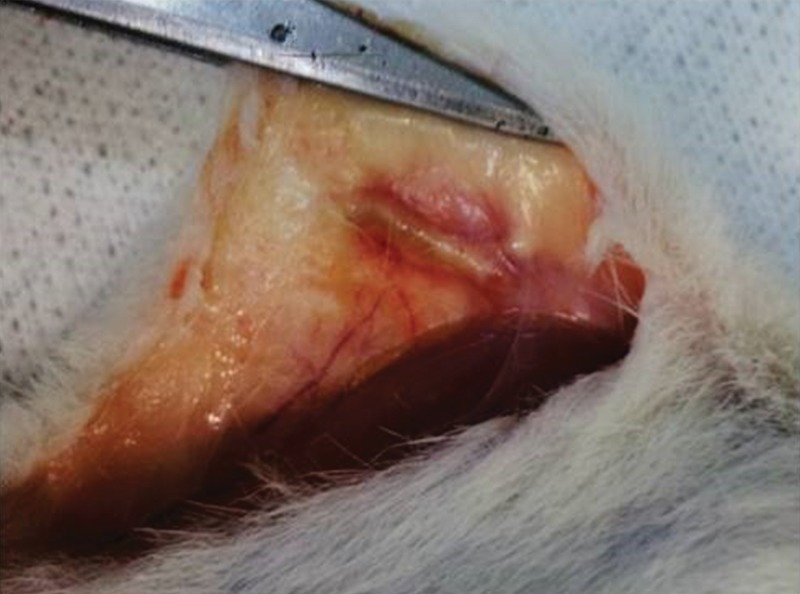
Syngeneic femur 15 days after implantation in the abdominal pocket.In the surgical picture, the subcutaneous tissue where the bone was positioned is swollen and hyperemic, and dilated blood vessels can be identified nearby. The bone is also mildly swollen and is fully encased by adherent inflammatory tissue (pseudocapsule). This foreign body-like reaction included multiple blood capillaries.

Figure 3 depicts the femur after 15 days of subcutaneous storage. The femur was swollen and fully covered by an inflammatory pseudocapsule. Both the diaphysis and epiphyses were widened. Handling of the graft confirmed that it had become more flexible because of fluid infiltration. Neither macroscopic bone nor bone marrow necrosis was prominent.

**Figure 4:**
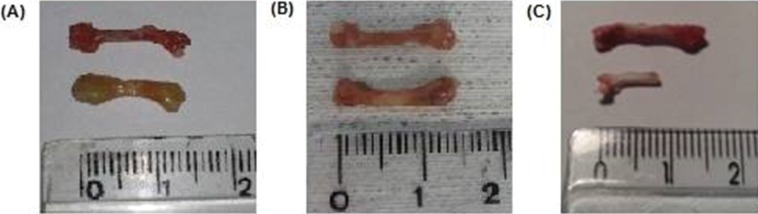
Pictures of control (upper image) and grafted femur (lower image) at different times. A: 15 days, B: 30 days, C: 60 days. It is perceived that, after 15 days, the implant is swollen and enlarged. By 30 days, the edema had disappeared and was replaced by bone dryness and brittleness. However, the structural integrity was mostly maintained. At the last follow-up (60 days), severe bone resorption and fragmentation in some cases permitted the recovery of as little as 50% of the syngeneic bone only. However, in most animals, bone destruction was not as advanced as in this picture.

After 30 and 60 days, the inflammatory tissue reaction around the bone continued to occur, although less hyperemia was observed. Bone edema disappeared and was replaced by progressive brittleness and resorption. By 60 days, bone resorption and fragmentation were conspicuous, and in certain animals, as little as 50% of the original femur could be recovered from the subcutaneous pouch. Bone marrow changes suggestive of necrosis were also noted at 30 days and were more remarkable after 60 days (Figures 2 and 4).

**Figure 5:**
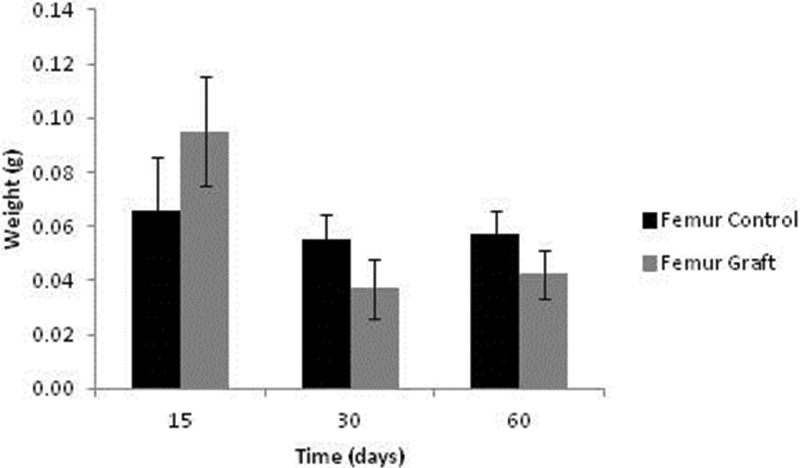
Femur weight after subcutaneous storage.

Bone weight data are presented in Figure 5. After 15 days, the grafts were heavier than the controls (*p*=0.038); however, these profiles were reversed after 30 and 60 days of implantation (*p*=0.035 and *p*=0.047, respectively).

**Figure 6:**
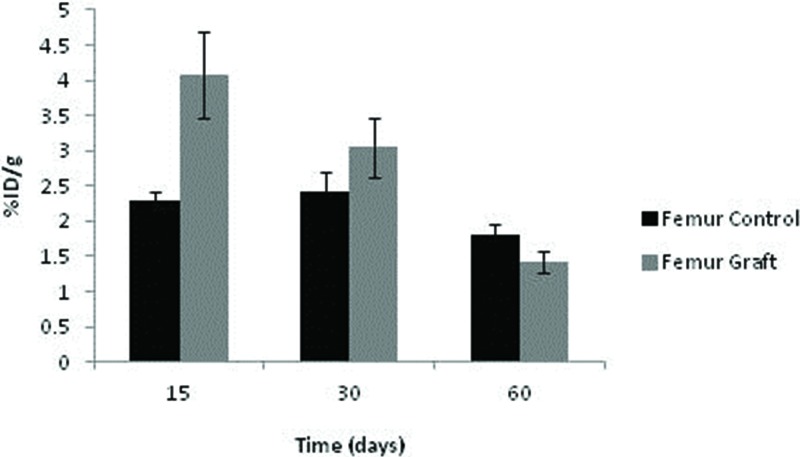
Uptake of ^99m^Tc-HYNIC-E-c(RGDfK)_2_ in the femur.

The uptake of ^99m^Tc-HYNIC-E-c(RGDfK)_2_ by the control femurs displayed some fluctuations depending on the time point, although no significant difference was noted. Among the grafts, the uptake was highest after 15 days (*p*=0.016) and subsequently diminished at 30 and 60 days (*p*=0.040 and *p*=0.043, respectively). Consequently, graft uptake exceeded that of controls under all circumstances (Figure 6).

**Figure 7:**
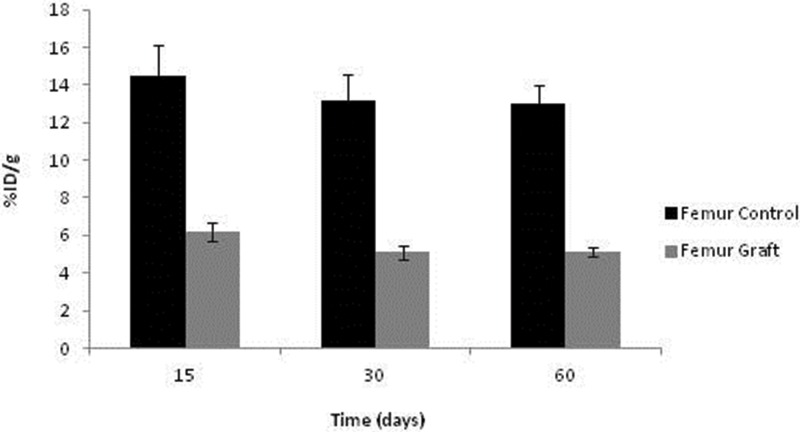
Uptake of ^99m^Tc-MDP in the femur.

No significant differences in ^99m^Tc-MDP were detected over time among the syngeneic implants or control femurs, although a tendency toward graft reduction at 30 and 60 days was evident. As expected, the control uptake was substantially higher than in the ischemic grafts. It should be emphasized that the avascular graft values were not negligible, indicating sustained viability and metabolic activity, as documented with ^99m^Tc-HYNIC-E-c(RGDfK)_2_ (Figure 7).

**Figure 8:**
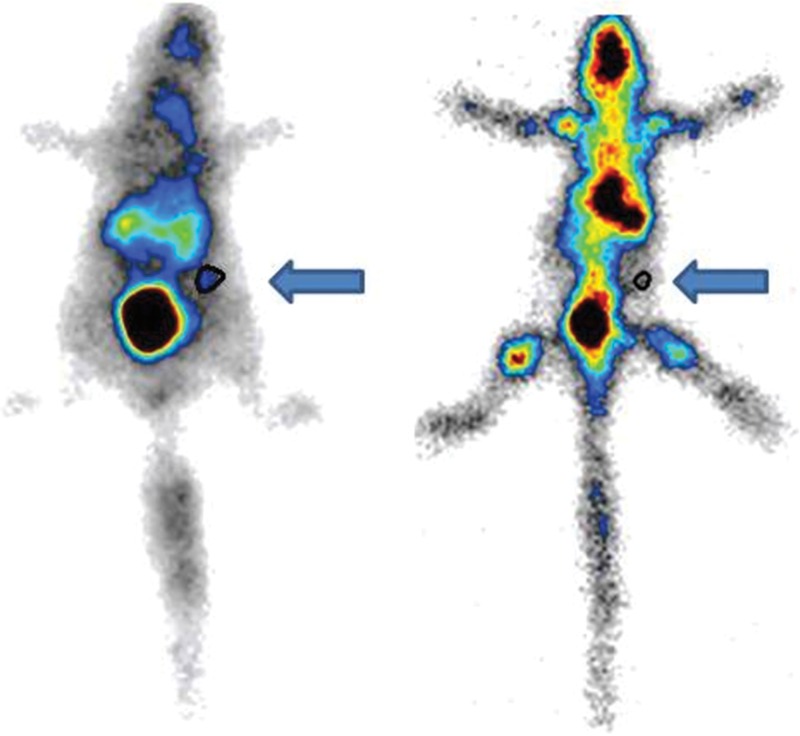
Gamma camera imaging of the femur graft after 15 days of abdominal storage. Left side (^99m^Tc-HYNIC-E-[c(RGDfK)_2_ region of interest (ROI= 0.5%) and right side ^99m^Tc-MDP uptake (ROI= 0.10%). The arrows indicate the implanted bone.

Static images obtained with a gamma camera (Figure 8) revealed the uptake of radiotracers in the entire body and in the femur grafts after 15 days.

**Figure 9:**
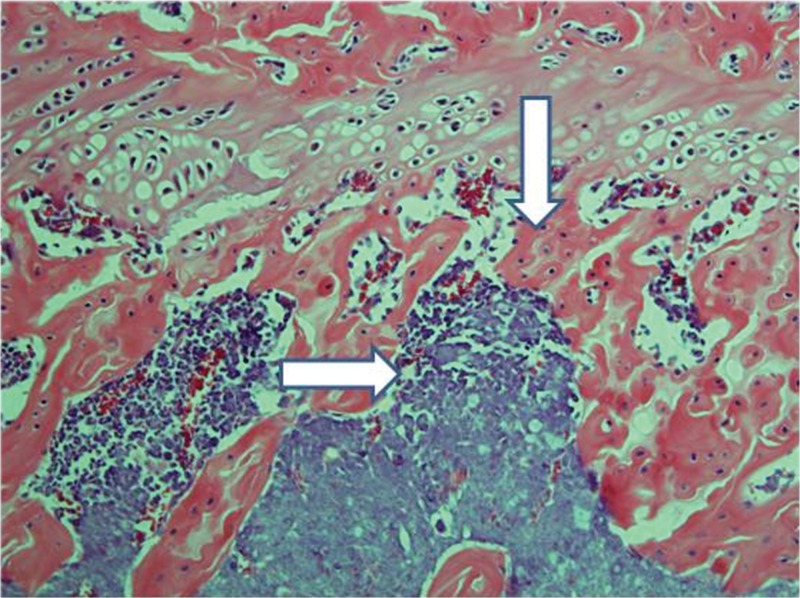
Control femur histology (H&E, 100 X): Bone trabeculae (vertical arrow) and bone marrow (horizontal arrow) were normal at all times, as expected.

**Figure 10:**
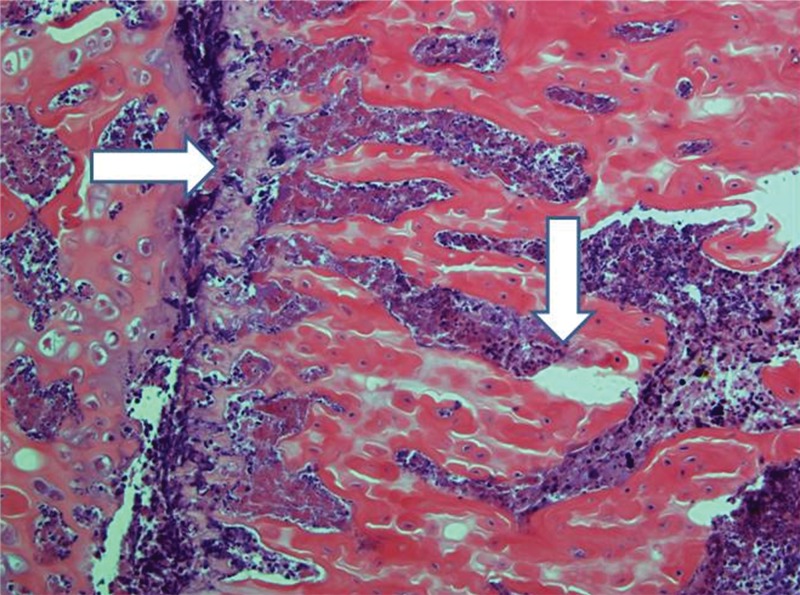
Implanted femur histology, 15 days (H&E, 100 X): The horizontal arrow indicates the foci of bone marrow necrosis, and the vertical arrow indicates an area of edema. Moderately altered bone trabeculae (not highlighted) could be identified.

Control femurs exhibited healthy trabeculae and bone marrow histology at all times, as expected (Figure 9). On ischemic slides after 15 days, mild marrow and bone edema could be identified, along with some degree of bone marrow necrosis (Figure 10).

**Figure 11:**
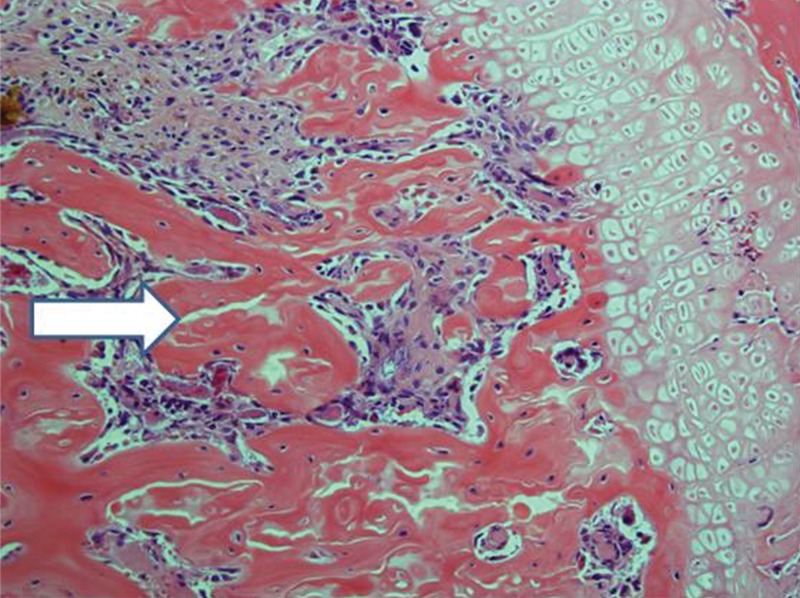
Implanted femur histology, 30 days (H&E, 100 X): Trabeculae are less sharp and more confluent, consistent with progressive bone deterioration (arrow). Edema is not prominent. Bone marrow histology varies from necrotic at some points to nearly intact at others.

**Figure 12:**
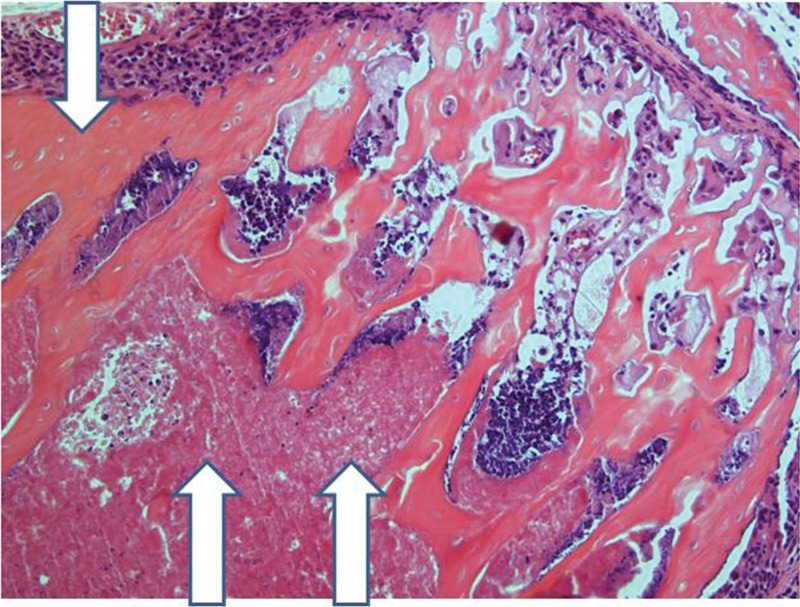
Implanted femur histology, 60 days (H&E, 100 X): Bone (upper arrow) and bone marrow (lower double arrows) degradation is more advanced. However, in keeping with the findings at 15 and 30 days, the pattern is not homogeneous, with seriously damaged areas, along with a better preserved femur.

After 30 days, edema was no longer conspicuous. Some areas exhibited bone resorption. The bone marrow did not change substantially compared with that at 15 days (Figure 11). By 60 days, areas exhibiting extensive destruction of the bone and bone marrow could be identified. Nevertheless, as at other time points, the femur showed a generally heterogeneous pattern. Depending on the anatomical location, areas that were in fairly good histological shape were identified, whereas other areas revealed severe damage (Figure 12).

## DISCUSSION

Osteonecrosis is a condition that results from the temporary or permanent loss of blood supply to the bone. Simultaneous direct damage to osteocytes, osteoclasts, bone marrow and other cells cannot be excluded in many circumstances, particularly during radiotherapy, alcohol use, drug use, or endotoxemia, potentially aggravating the consequences of ischemia. Indeed, some studies have reported that this difficult-to-define process is more related to apoptosis than to necrosis 14.

Osteonecrosis has also been designated as avascular, aseptic, ischemic, subchondral, and subarticular necrosis. The last two designations refer to its tendency to attack the epiphyseal regions of long bones Although it might be completely asymptomatic, when osteonecrosis occurs in implanted bone, loss of function with permanent disability or malfunction of the graft is inevitable after major insult unless the bone can regenerate and rebuild itself spontaneously or recover via an intervention 1,2,14.

The first report of drug-related osteonecrosis appeared in 1845 15. At this time, matches were entering the world market, and all were made with yellow phosphorus. Prolonged exposure to this chemical, especially by workers who manufactured matches, could trigger extensive and disfiguring necrosis of the mandible. Decades later, yellow phosphorus was banned, and other molecules were selected for commercial use.

Calve Legg Perthes disease was first recognized in 1910 16, and with the progress in orthopedics after World War II, many investigations into osteonecrosis ensued 1,2,14.

Clinical diagnostic tools for bone loss do not distinguish among bone matrix turnover changes, cell apoptosis, and tissue necrosis. Resources include biochemical bone turnover markers, such as serum C-telopeptide (CTX), urine N-telopeptide (NTX), serum bone-specific alkaline phosphatase (BALP), serum osteocalcin, and serum procollagen type 1 N-terminal propeptide (PINP). The first two reflect bone resorption, whereas the latter three are relevant to bone formation 17,18.

Similarly, photon and X-ray absorptiometry for bone densitometry depicts the end results of all remodeling phenomena occurring within the skeleton mass, independent of their nature 19.

Conventional CT and MRI can accurately identify anatomical changes, such as localized erosion and bone loss, and they can provide clues about necrotic and healthy zones. Nevertheless, only cumbersome and specialized techniques, such as micro-CT, micro-MRI, and functional MRI, permit making inferences about cell metabolism, disease progression, tissue healing, and other functional processes. Limited information is available, and no investigation has yet addressed osteonecrosis or angiogenesis 20,21,22.

Tissue morphofunctional analysis, namely, the histology and histopathology of bone tissue, is a disease-specific, precise and reliable technique. It clearly identifies the involvement of bone matrix and various cell lineages in ongoing changes. This tool was employed here for confirmation purposes. However, its clinical applications are restricted because this analysis is a highly invasive, costly, and time-consuming procedure.

In the current protocol, the diagnosis of osteonecrosis and angiogenesis was conducted by means of radiotracers and bone imaging. The terms “osteonecrosis” and “angiogenesis” are employed rather nonspecifically as synonyms of bone resorption and the restoration of tissue viability, respectively, because receptor measurements and the quantification of genetic and molecular markers are not included in the protocol. The study results were collected using a classic technique. Technetium-99m methylene diphosphonate (^99m^Tc-MDP) bone scintigraphy has long been used for the evaluation of normal bone and bone lesions by chemisorption, followed by hydroxyapatite exchange with the inorganic matrix of the bone 23,24.

Angiogenesis, which is the process of neovasculature formation from pre-existing blood vessels, is widely considered to be essential to ensure the nutrient and oxygen supply to ischemic tissues 25. No previous reports addressing the direct or indirect assessment of angiogenesis in avascular bone were found in the literature; thus, to the best of our knowledge, this work is the first attempt at such an evaluation.

Angiogenesis involves many mediators, including integrins. The extracellular domains of many integrins recognize the RGD (Arg-Gly-Asp) tripeptide found in several extracellular macromolecules, such as fibronectin, vitronectin, fibrinogen and osteopontin 11. This fact was the rationale underlying the selection of ^99m^Tc-HYNIC-E-c(RGDfK)_2_ as an additional radiotracer in this study_._

It should be noted that αvβ3 integrin is expressed in osteoclasts, which are bone-resorbing cells, and it binds to some RGD-containing proteins, including osteopontin, which is abundant in bone. The use of RGD radiotracers has been experimentally reported in the form of PET-CT for the identification of osteoclasts 26.

However, RGD is considered a promising agent for only osteoclast-related diseases, such as Paget's disease or rheumatoid arthritis. Osteoclast activity has been reported in subchondral bone, such as human osteonecrotic femoral heads, with increased osteoblast expression found in neighboring areas 27. Avascular bone necrosis is not listed among osteoclast-related diseases, but overlap between angiogenesis and osteoclast affinity might interfere with the interpretation of the findings.

For this reason, among others, histological confirmation was sought to shed light on the potentially conflicting affinities of ^99m^Tc-HYNIC-E-c(RGDfK)_2_ in this setting.

Experience with abdominal subcutaneous autologous flaps of calvarial bone stored for periods of two months or more in the context of severe intracranial hypertension may be pertinent to the current model, although cancellous bone is prominent in the skull and not in cortical tissue 3,4,28.

Singla et al. (2014) 12 observed angiogenesis in 14% of patients. No relationship was noted between angiogenesis and the duration of bone flap preservation. Six percent of the bone flaps showed new bone formation. All of the bone flaps with new bone formation had underlying osteoblastic activity. The bone implants were maintained in the subcutaneous pouch for a highly variable period, usually between 3 and 12 months.

New bone generation was occasionally detected on histological slides in this experience; however, bone and bone marrow damage constituted the most noticeable features. Nevertheless, one should emphasize the heterogeneity of the results, which was not perceived in the figures in the article because representative images were selected. At all of the follow-ups, but especially after 30 and 60 days, quite healthy areas were prominent, in contrast with heavily compromised skeleton tissue. This finding could be another limitation of the histological monitoring of bone necrosis because the interpretation of biopsy material would become dependent on regional fluctuations in tissue damage.

The same morphological heterogeneity prevented point-by-point correlations between histology and radiotracer profiles, which might elucidate nuclear marker affinities in the ongoing necrosis phenomenon regarding angiogenesis, new bone synthesis, and osteoclastic resorption. Nevertheless, the tendency toward progressive deterioration in femur histology from 15 to 60 days was fully compatible with similar results with ^99m^Tc-MDP, which is a nonspecific bone health marker. At the same time, the maintenance of acceptable bone viability despite full ischemia, notably by 15 and 30 days but decaying afterwards, could be easily explained by active angiogenesis during the early periods, as suggested by the ^99m^Tc-HYNIC-E-c(RGDfK)_2_ uptake curve.

It should be noted that *ex vivo* isotopic samples were selected here, which is something that is impractical in patient studies; however, it was only used for greater accuracy of the experiment. The available clinical scanners provide sufficient resolution for both purposes envisaged in the design, namely, the uptake measurements and imaging studies 9,11,24,29.

There are reasons to believe that periosteal, endosteal, and medullary cells within 0.2 mm of the bone surface survive after excision and bone implantation, while most others succumb 30,30. Moreover, subcutaneous tissue is anatomically a poorly vascularized bed. Until pouch vasodilatation and graft angiogenesis occur, which can take days or weeks, cell death could be inevitable, potentially hampering the future viability of the graft.

Given that only modified imaging methods, and not conventional methods 20,21,22, have been deemed suitable for bone metabolism and viability exploration, there could be an advantage within this context of considering isotopic techniques. These procedures were intrinsically functional and global, depicting all of the anatomical segments targeted by systemically injected biomarkers. Infarcted areas were naturally excluded because radionuclide uptake becomes negligible 29.

The protocol was successful, and a simple, stable and convenient model was created. At the same time, the two radiotracers proved easy to employ, and the results were encouraging. Even in fully ischemic tissue, devoid of any anatomically recognized blood flow, both were clearly taken up and could be identified by imaging.

These are comparatively inexpensive and available agents, at least in institutions with access to nuclear medicine facilities. Technetium has a chemical half-life of 6 hours and a biological half-life of 24 hours. Radiotracer studies can be safely repeated as often as every week, if clinically indicated. Radiotracers deserve investigation in bone grafts and ischemic bone disease, among other settings, not only in cases of the abdominal preservation of cranial vault grafts with the purpose of expanding the current findings but also in unearthing target-specific biomolecular processes connected to necrotic initiation, progression and resolution.

## CONCLUSIONS

1. A new experimental model of bone osteonecrosis in mobile animals was created, which was convenient and practical, even for relatively long follow-up periods.
2. The diagnosis of graft conditions by systemic radiotracers was demonstrated for the first time in a totally devascularized major bone structure, thus paving the way for future studies in additional settings.
3. The histological heterogeneity of bone and bone-marrow necrosis suggested that global noninvasive functional bone assessment provided by the isotopes could exceed the advantages of bone biopsy and other more complex alternatives.
